# Regulatory Effects of Metformin, an Antidiabetic Biguanide Drug, on the Metabolism of Primary Rat Adipocytes

**DOI:** 10.3390/molecules27165250

**Published:** 2022-08-17

**Authors:** Tomasz Szkudelski, Klaudia Konieczna, Katarzyna Szkudelska

**Affiliations:** Department of Animal Physiology, Biochemistry and Biostructure, Poznan University of Life Sciences, Wołynska 35, 60-637 Poznan, Poland

**Keywords:** metformin, adipocytes, lipogenesis, glucose transport, lipolysis, adrenergic receptor pathway, adenosine A_1_ receptor pathway, PKA, glucose, alanine, lactate

## Abstract

Metformin is a biguanide compound commonly applied in humans with type 2 diabetes. The drug affects different tissues, including fat tissue. The direct influence of metformin on cells of fat tissue, i.e., adipocytes, is poorly elucidated. In the present study, the short-term (4-h) effects of metformin on lipogenesis, glucose transport, lipolysis, and lactate release in primary rat adipocytes were explored. It was demonstrated that metformin reduced insulin-induced lipogenesis and increased glucose transport into adipocytes. The tested compound also decreased lactate release from fat cells. It was shown that metformin substantially limited lipolysis stimulated by epinephrine (adrenergic receptor agonist) and dibutyryl-cAMP (direct activator of protein kinase A). Moreover, metformin decreased the lipolytic process triggered by DPCPX (adenosine A_1_ receptor antagonist). In the case of each lipolytic stimulator, the drug evoked a similar inhibitory effect in the presence of 3 and 12 mM glucose. The lipolytic response of adipocytes to epinephrine was also found to be reduced by metformin when glucose was replaced by alanine. It was demonstrated that the tested compound limits the release of both glycerol and fatty acids from fat cells. The results of the present study provided evidence that metformin significantly affects the metabolism of primary rat adipocytes. Its action covers processes related to lipid accumulation and release and occurs after relatively short-term exposure.

## 1. Introduction

Diabetes is a grave metabolic disease, affecting a large number of people worldwide. Currently, the disease affects about 10% of adults, and morbidity is increasing. Diabetes is divided into many types. Type 2 diabetes is the most common and covers about 90% of all cases. Humans with type 2 diabetes manifest defective insulin secretion and action and are often overweight or obese. Impaired insulin action affects the main insulin-sensitive tissues, i.e., liver, skeletal muscle, and fat tissue. Type 2 diabetes develops slowly, and blood glucose levels are relatively mildly elevated compared with type 1 diabetes. However, sustained hyperglycemia largely contributes to a progressive failure of pancreatic β-cells [1,2]. Such hyperglycemia is also associated with oxidative and inflammatory stress, which impairs insulin action additionally [3,4,5]. Moreover, over time, humans with type 2 diabetes develop diabetic complications [1,2].

Adipose tissue dysfunction has a relevant role in the pathogenesis and progression of type 2 diabetes. Under physiological conditions, fat tissue stores energy and secretes multiple hormones with regulatory functions [6,7]. However, excessive adipose tissue accumulation leads to impaired glucose homeostasis, insulin resistance, and type 2 diabetes. Disturbed secretion and action of adipocyte-derived hormones, predominantly adiponectin, leptin, and resistin, also impairs insulin action [3,8,9]. Moreover, obesity is strongly associated with the release of proinflammatory cytokines, which additionally worsens insulin sensitivity [10,11].

In humans with type 2 diabetes, a few classes of drugs are used to effectively treat the disease [1,2,3]. Metformin belongs to the biguanide group and is a blood-glucose-lowering compound without risks of hypoglycemia. The alleviation of hyperglycemia by metformin and other drugs is pivotal to limiting diabetes progression. The liver is thought to be the main target for metformin. The drug reaches liver cells and suppresses hepatic glucose output, which leads to reduced blood glucose levels. However, other insulin-sensitive tissues, including adipose tissue, could also be affected by this compound. Metformin therapy is known to be associated with numerous beneficial effects on fat tissue. One of relevance is improved insulin sensitivity in adipose tissue [12,13]. Metformin reduces adiposity and positively affects adipocyte energy metabolism. Moreover, the drug upregulates the expression of uncoupling protein-1 in fat tissue [14]. Metformin treatment was also shown to alleviate interstitial fibrosis of adipose tissue. This effect is beneficial since it is associated with increased insulin sensitivity [12]. Many effects of metformin on adipose tissue are known to be mediated by AMP-activated protein kinase (MAPK). AMPK is an intracellular energy sensor, and its activation affects processes related to energy metabolism. This enzyme is phosphorylated and activated in adipose tissue in response to metformin therapy [12]. It is well established that obesity is associated with chronic adipose tissue-related low-grade inflammation. This pathological condition impairs insulin sensitivity and plays a relevant role in the pathogenesis of type 2 diabetes. However, metformin is capable of reducing adipose tissue-related inflammation [14]. Moreover, beneficial effects on insulin action following metformin treatment are related to increased production and secretion of adiponectin. This is one of the relevant adipocyte-derived hormones that improves insulin sensitivity. Reduced blood adiponectin levels largely contribute to excessive tissue lipid accumulation and the resulting insulin resistance while restoration of appropriate concentrations of adiponectin has the opposite effect [12,13].

The results of in vitro studies provided evidence that metformin has been implicated in the regulation of fat cell biology. Exposure to metformin affects fat cell metabolism, maturation, gene expression, and many other processes. One of the relevant effects is the inhibition of adipogenesis. This is due to the metformin-induced downregulated expression of genes related to adipogenesis and obesity [15,16,17]. Moreover, exposure to metformin limits preadipocyte maturation and lipid accumulation in 3T3-L1 cells [18]. The effects of the drug on adipogenesis may be, however, dual, depending on its concentration. It was revealed that metformin promotes adipogenesis at lower concentrations and inhibits this process at higher amounts [16]. Moreover, metformin was shown to ameliorate fibrosis in insulin-resistant and hypertrophied adipocytes. This effect largely contributes to better insulin sensitivity [19]. Metformin may also promote glucose transport into fat cells, predominantly via the glucose transporter GLUT4 [20]. However, other studies demonstrated that the drug is effective solely in the presence of insulin and fails to affect basal glucose uptake [21,22]. In line with the results of in vivo studies, some effects of metformin on fat cells are mediated by AMPK. Metformin was shown to activate AMPK in 3T3-L1 cells [23] and human adipocytes [24]. It was also demonstrated that fat cell exposure to the drug may affect the function of mitochondria. Metformin is capable of ameliorating insulin resistance related to genetically induced mitochondrial dysfunction in 3T3-L1 adipocytes [25]. This compound also inhibits mitochondrial respiration in 3T3-L1 adipocytes [26] and diminishes oxygen consumption by human preadipocytes and adipocytes [24]. These results indicate that metformin is capable of affecting fat cells by changes in AMPK activity and mitochondrial metabolism.

It should be noted that some effects of metformin action on isolated cells have not been confirmed in humans receiving this drug. Moreover, some beneficial changes evoked by the drug, such as improved insulin sensitivity, may appear without a detectable reduction in body weight or adiposity. This is possible since it may be related to changes at the cellular level involving the insulin signaling pathway, i.e., better phosphorylation of signaling proteins.

The majority of data related to metformin action on fat cells is derived from experiments with 3T3-L1 cells. These cells differ markedly from freshly isolated adipocytes. Additionally, studies addressing preadipocyte maturation and gene expression need long-term exposure to the tested compound, compared with metabolic research. Thus, the direct effects of metformin on adipocyte metabolism have been poorly explored. Moreover, some controversy in the literature related to its action can be found. This is mainly due to differences in experimental conditions, such as the metformin concentrations, time of exposure, and the kind of cells used in the study (freshly isolated or cell lines). Such differentiation may result not only in the diverse effects of metformin but also in various mechanisms of its action [13,16,21,22,24]. The present study aimed to determine the short-term effects of metformin on lipogenesis, glucose transport, lipolysis, and lactate release in freshly isolated rat adipocytes. These processes are highly relevant since they not only affect adipocytes but also other kinds of cells [27,28]. The mechanism underlying metformin action has also been proposed.

## 2. Results

### 2.1. Effects of Metformin on Lipogenesis

**Figure 1:**
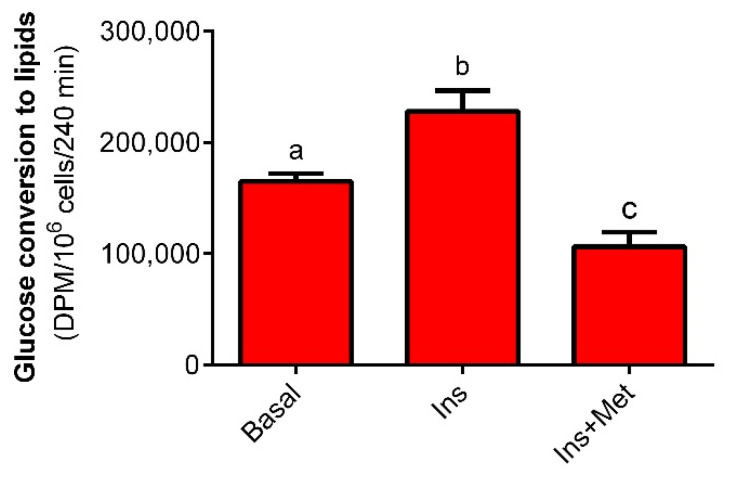
The effects of metformin on glucose conversion to lipids in isolated rat adipocytes. Adipocytes were incubated in a buffer containing 3 mM glucose, glucose D-[^14^C(U)], 3% BSA, and 10 mM HEPES. Adipocytes were incubated without insulin (basal lipogenesis) or in the presence of 10 nM insulin (stimulated lipogenesis). To study the effects of metformin on insulin-induced lipogenesis, cells were incubated with insulin and 1 mM metformin. After the end of the incubation, total lipids derived from glucose were extracted, and their radioactivity was measured. Ins—insulin, Met—metformin, DPM—disintegrations per minute. The obtained results represent the mean ± SEM from 3 independent experiments in 5 repetitions (*n* = 15). Values marked by different letters differ significantly at *p* < 0.05.

It was shown that 4-h exposure of isolated rat adipocytes to 10 nM insulin was associated with increased glucose conversion to total lipids. Insulin increased lipogenesis by 40%, compared to the basal process, and the effect was statistically significant. However, insulin-stimulated lipogenesis was markedly decreased in the presence of 1 mM metformin. The tested drug reduced lipogenesis by 55%, and its effect was statistically significant (Figure 1).

### 2.2. Effects of Metformin on Glucose Transport

**Figure 2:**
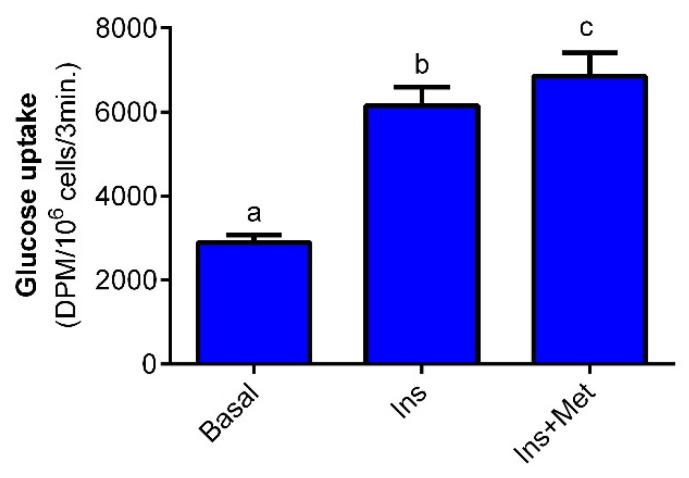
The effects of metformin on glucose transport into isolated rat adipocytes. Adipocytes were incubated in buffer containing 0.5 mM glucose, 2-deoxy-D-glucose-[1-^14^C], 3% BSA, and 10 mM HEPES. First, cells were preincubated for 10 min without metformin or in the presence of 1 mM metformin. Then, 10 nM insulin was added to both groups (Ins and Ins + Met). The additional group of cells (Basal) was preincubated with 0.5 mM glucose without insulin. After 20 min, 2-deoxy-D-glucose-[1-^14^C] was added and incubations were performed for 3 min. Finally, the radioactivity derived from 2-deoxy-D-glucose-[1-^14^C] into adipocytes was measured. Ins—insulin, Met—metformin, DPM—disintegrations per minute. The obtained results represent the mean ± SEM from 3 independent experiments in 5 repetitions (*n* = 15). Values marked by different letters differ significantly at *p* < 0.05.

We demonstrated that 10 nM insulin increased glucose transport into isolated rat adipocytes, compared to the basal values. This effect was 110% and was statistically significant. The combination of insulin and 1 mM metformin resulted in an additional rise in glucose transport compared to the effects elicited by insulin alone. In the present study, metformin increased glucose transport by 15%, and the observed effect was statistically significant (Figure 2).

### 2.3. Effects of Metformin on Glycerol Release

**Figure 3:**
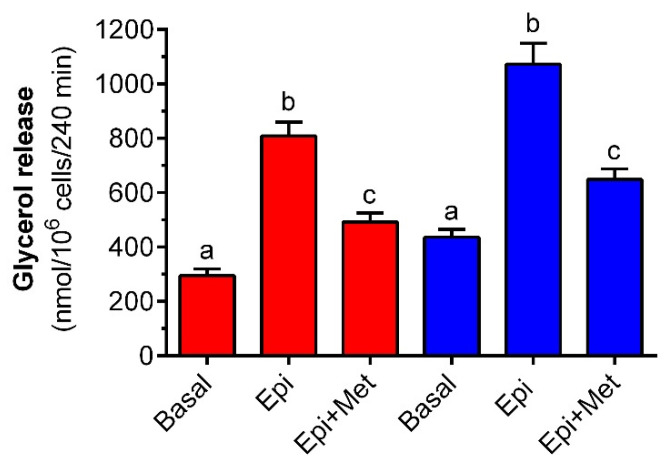
The effects of metformin on epinephrine-induced glycerol release in isolated rat adipocytes in the presence of low and high concentrations of glucose. Adipocytes were preincubated for 3 h with or without 1 mM metformin in the buffer containing 3 mM glucose (red bars) or 12 mM glucose (blue bars). Then, 0.5 µM epinephrine was added to all cells (except for basal) and incubations were performed for an additional 1 h. After the end of the incubations, adipocytes were removed and the glycerol content in the buffer was determined. Epi—epinephrine, Met—metformin. The obtained results represent the mean ± SEM from 3 independent experiments in 5 repetitions (*n* = 15). Values marked by different letters within each experimental group differ significantly at *p* < 0.05.

As expected, exposure of primary rat adipocytes to 0.5 µM epinephrine significantly increased glycerol release compared with the basal values. In the presence of 3 mM glucose, epinephrine augmented glycerol release by 180%. However, the lipolytic response to the hormone was significantly blunted in fat cells exposed to 1 mM metformin. The tested drug significantly reduced epinephrine-induced glycerol release by 60% (Figure 3). In adipocytes maintained in the buffer containing 12 mM glucose, the mean epinephrine-stimulated glycerol output was increased by 145% compared with the appropriate control. This change was also statistically significant. It was also shown that metformin decreased glycerol release from fat cells incubated with epinephrine and 12 mM glucose. This effect was 65% and was statistically significant (Figure 3).

**Figure 4:**
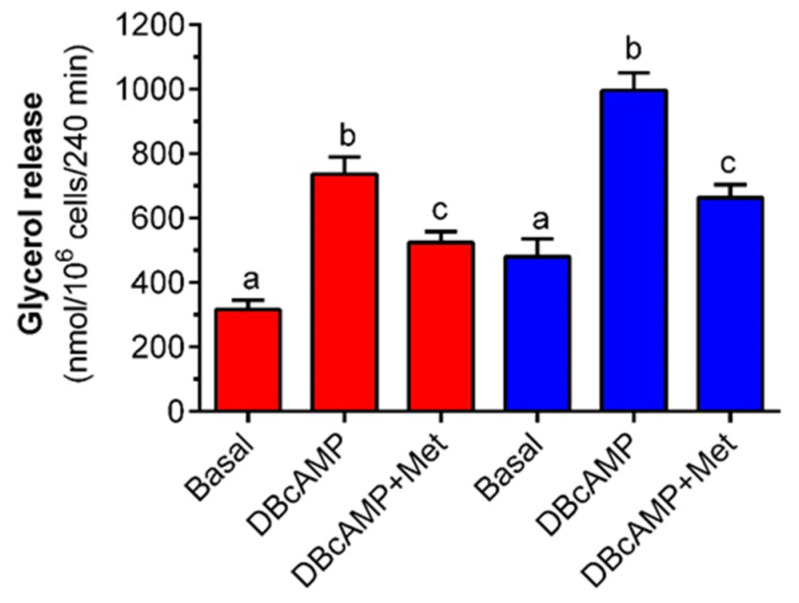
The effects of metformin on DB-cAMP-induced glycerol release in isolated rat adipocytes in the presence of low and high concentrations of glucose. Adipocytes were preincubated for 3 h with or without 1 mM metformin in the buffer containing 3 mM glucose (red bars) or 12 mM glucose (blue bars). Then, 0.5 mM DB-cAMP was added to all cells (except for basal) and incubations were performed for an additional 1 h. After the end of the incubations, adipocytes were removed and the glycerol content in the buffer was determined. DB-cAMP—dibutyryl-cAMP, Met—metformin. The obtained results represent the mean ± SEM from 3 independent experiments in 5 repetitions (*n* = 15). Values marked by different letters within each experimental group differ significantly at *p* < 0.05.

Similar to the effects of epinephrine, adipocyte exposure to 0.5 mM DB-cAMP evoked a clear-cut increase in glycerol output compared with non-stimulated cells. In the presence of 3 mM glucose, DB-cAMP elevated the glycerol content in the incubation medium by 130%, and the observed effect was statistically significant. Exposure of fat cells to metformin substantially decreased DB-cAMP-induced glycerol output. Metformin limited glycerol release by 70% and its influence was statistically significant (Figure 4). Moreover, it was shown that glycerol release triggered by DB-cAMP in the presence of 12 mM glucose was augmented compared with control values. The rise was 110% and was statistically significant. However, exposure of isolated adipocytes to metformin decreased DB-cAMP-stimulated glycerol release. The inhibitory effect was statistically significant and reached 60% of the values noted without metformin (Figure 4).

**Figure 5:**
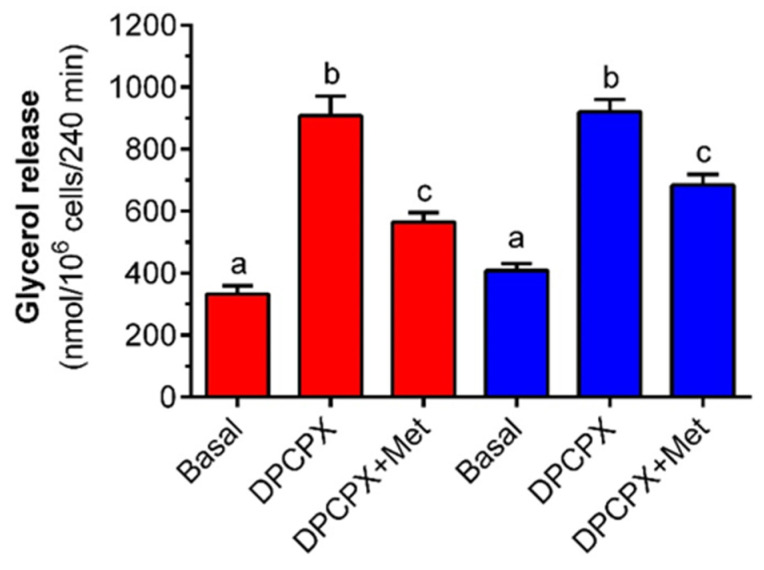
The effects of metformin on DPCPX-induced glycerol release in isolated rat adipocytes in the presence of low and high concentrations of glucose. Adipocytes were preincubated for 3 h with or without 1 mM metformin in the buffer containing 3 mM glucose (red bars) or 12 mM glucose (blue bars). Then, 0.5 µM DPCPX was added to all cells (except for basal) and incubations were performed for an additional 1 h. After the end of the incubations, adipocytes were removed and the glycerol content in the buffer was determined. DPCPX—8-Cyclopentyl-1,3-dipropylxanthine, Met—metformin. The obtained results represent the mean ± SEM from 3 independent experiments in 5 repetitions (*n* = 15). Values marked by different letters within each experimental group differ significantly at *p* < 0.05.

It was also revealed that adipocyte exposure to 0.5 µM DPCPX led to a marked elevation in glycerol output to the incubation medium. The observed rise in the case of adipocytes maintained in buffer with 3 mM was 175% and was statistically significant. Exposure to metformin was, however, associated with a clear-cut anti-lipolytic effect. Under these experimental conditions, metformin diminished glycerol release by 60% and its effect was statistically significant (Figure 5). Incubations of isolated adipocytes with DPCPX in the presence of 12 mM glucose evoked a marked increase in glycerol release compared with the appropriate control. The cellular response to DPCPX stimulation was 125% and was statistically significant. Metformin was found to effectively reduce glycerol release induced by DPCPX in the presence of 12 mM glucose. The tested drug lowered the glycerol content in the incubation medium by 75%, and its action was statistically significant (Figure 5).

**Figure 6:**
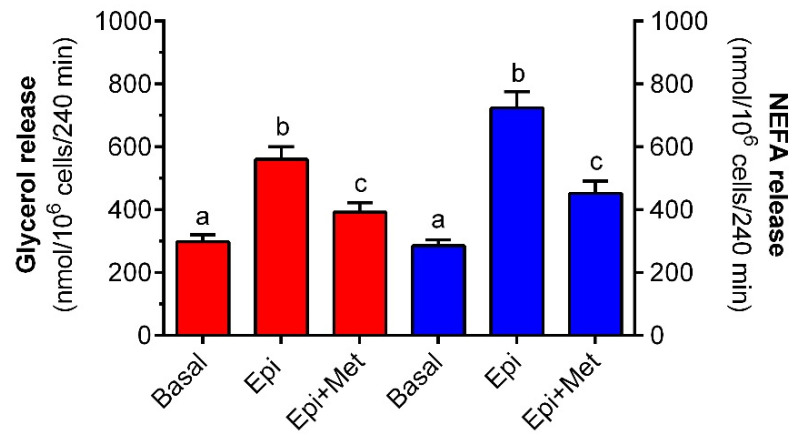
The effects of metformin on epinephrine-induced glycerol and NEFA release in isolated rat adipocytes in the presence of alanine or glucose. Adipocytes were preincubated for 3 h with or without 1 mM metformin in the buffer containing 12 mM alanine (red bars) or 3 mM glucose (blue bars). Then, 0.5 µM epinephrine was added to all cells (except for basal) and incubations were performed for an additional 1 h. After the end of the incubations, adipocytes were removed and the glycerol or NEFA content in the buffer was determined. Epi—epinephrine, Met—metformin, NEFA—non-esterified fatty acid. The obtained results represent the mean ± SEM from 3 independent experiments in 5 repetitions (*n* = 15). Values marked by different letters within each experimental group differ significantly at *p* < 0.05.

Epinephrine-promoted glycerol release in the presence of 12 mM alanine was shown to be markedly enhanced compared with incubations without the hormone. The rise reached 90% and was statistically significant. In adipocytes incubated in the presence of alanine, metformin exposure was associated with reduced epinephrine-induced glycerol release. Under these experimental conditions, the glycerol output was lower by 70%, and the observed change was statistically significant (Figure 6).

### 2.4. Effects of Metformin on NEFA Release

In the present study, epinephrine and metformin were shown to substantially affect NEFA release from freshly isolated rat adipocytes. The effects of both compounds were the opposite. It was demonstrated that epinephrine augmented NEFA release from adipocytes incubated in the presence of 3 mM glucose. NEFA release induced by the hormone was higher than basal by 155% and was statistically significant. Metformin was found to exert an inhibitory effect on NEFA release in the presence of epinephrine. In adipocytes subjected to the tested drug, the mean NEFA concentration in the incubation buffer was reduced by 65% and the effect was statistically significant (Figure 6).

### 2.5. Effects of Metformin on Lactate Release

**Figure 7:**
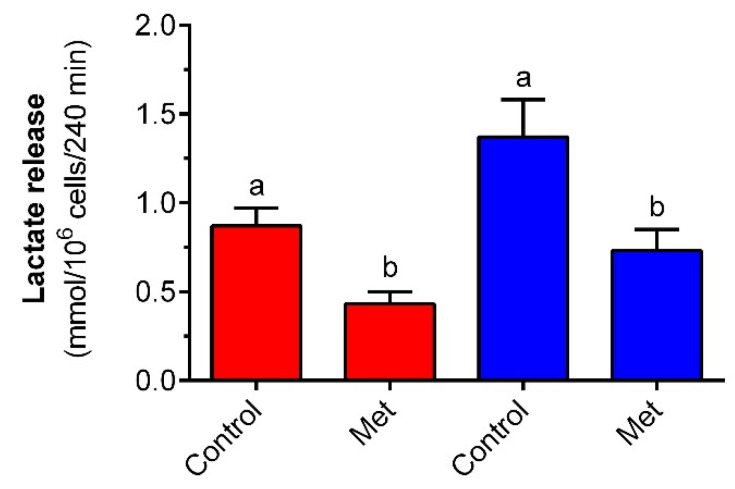
The effects of metformin on lactate release from isolated rat adipocytes in the presence of low and high concentrations of glucose. Adipocytes were incubated for 4 h with or without 1 mM metformin in buffer containing 3 mM glucose (red bars) or 12 mM glucose (blue bars). After the end of the incubations, adipocytes were removed and the lactate content in the buffer was determined. Epi—epinephrine, Met—metformin. The obtained results represent the mean ± SEM from 3 independent experiments in 5 repetitions (*n* = 15). Values marked by different letters within each experimental group differ significantly at *p* < 0.05.

Adipocyte exposure to low and high concentrations of glucose was shown to be associated with differences in lactate release. Fat cells subjected to 12 mM glucose released more lactate compared with values revealed in the presence of 3 mM glucose. The rise was 60% and was statistically significant. Metformin effectively reduced lactate output by isolated rat adipocytes. Exposure of fat cells to metformin in the presence of 3 mM glucose diminished lactate release by half and the effect was statistically significant. In adipocytes maintained in the buffer with 12 mM glucose, the tested drug decreased lactate output by 55% in a statistically significant way (Figure 7).

### 2.6. Effects of Metformin on Adipocyte Viability

**Figure 8:**
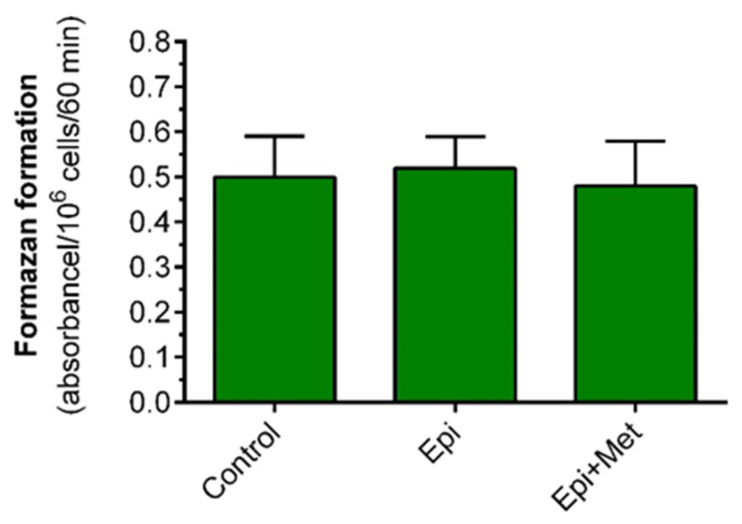
The effects of metformin on the viability of isolated rat adipocytes. Adipocytes were preincubated for 3 h with or without 1 mM metformin in buffer containing 3 mM glucose. Then, 0.5 µM epinephrine or 1 mM metformin was added and incubations were performed for an additional 1 h. Then, all cells were washed with the buffer without metformin and were incubated in the buffer containing 3 mM glucose with 0.5 mg/mL MTT. After 1 h of incubation, isopropanol was added, and tubes were shaken and centrifuged. Afterward, the formazan content was determined. Epi—epinephrine, Met—metformin, MTT—3-(4,5-dimethylthiazol-2-yl)-2,5-diphenyltetrazolium bromide. The obtained results represent the mean ± SEM from 3 independent experiments in 5 repetitions (*n* = 15).

We showed that incubations of primary adipocytes with epinephrine or epinephrine with metformin were not associated with marked changes in cell viability. The mean MTT absorbance, reflecting formazan formation, in fat cells exposed to epinephrine was increased by only 5%. Absorbance measured in the case of adipocytes subjected to epinephrine and metformin was decreased by 8% compared with non-treated cells. In each case, these differences were not statistically significant (Figure 8).

## 3. Discussion

We showed that metformin markedly affects the metabolism of primary rat adipocytes. Lipogenesis and lipolysis are pivotal processes associated with lipid synthesis and decomposition, respectively. It was demonstrated that metformin decreases glucose conversion to lipids. The first step of this process is intracellular glucose transport, followed by glucose metabolism to lipids. Reduced lipogenesis may result from decreased glucose transport and/or changes in intracellular metabolism. To better elucidate this issue, the effects of metformin on glucose transport were explored. It was revealed that metformin increased glucose transport into rat adipocytes. This is in line with studies showing that metformin (1 mM, 24-h exposure) increases glucose transport into human adipocytes [29]. Similar stimulatory effects of metformin (1 mM, 96 h exposure) were shown in rat adipocytes [30]. These results clearly indicate that decreased lipogenesis evoked by metformin is not due to reduced glucose transport but results from changes in glucose metabolism.

It was shown that the 4-h incubation of rat adipocytes with metformin attenuates the lipolytic response to epinephrine. Lipolysis induced by epinephrine is preceded by the sequence of events covering hormone binding to the adrenergic receptor, stimulation of Gs proteins, activation of adenylate cyclase, a rise in intracellular cAMP levels, activation of protein kinase A (PKA) and intracellular lipases, and phosphorylation of perilipins. This is followed by TG hydrolysis and the release of glycerol and NEFA from fat cells [6]. Similar inhibitory effects of metformin on lipolysis triggered by isoproterenol were found in human [31,32] and rat [33] adipocytes. Other studies have revealed that long-term (24-h) exposure of human fat cells to metformin reduced lipolysis stimulated by forskolin (activator of adenylate cyclase), isoproterenol, IBMX (adrenergic receptor agonists), and various inflammatory agents [2]. The inhibitory effects of metformin on lipolysis induced by isoproterenol and tumor necrosis factor-α (TNF-α) were shown in rat adipocytes after 24-h incubation [34]. The anti-lipolytic effect of the tested drug may result from changes at different steps of the lipolytic cascade or may be due to its influence on other lipolysis-related events. To better elucidate this issue, epinephrine was replaced by DB-cAMP, which is the direct activator of PKA. DB-cAMP enhances lipolysis, with the omission of the steps before PKA. It was shown that metformin limits the lipolytic process stimulated by DB-cAMP, and the effect is similar to inhibition in the presence of epinephrine. This indicates that metformin action in the adrenergic pathway is not upstream of PKA.

Apart from epinephrine, lipolysis is also regulated by adipocyte-derived adenosine. Adenosine is continuously released from fat cells, binds to its membrane receptor, and suppresses the lipolytic process. However, a blockade of the adenosine A_1_ receptor is associated with the elevated release of glycerol and NEFA [6,35]. It is also well established that abnormal signaling via the adenosine A_1_ receptor pathway contributes to some metabolic diseases [36]. Given the great relevance of this regulation, the influence of metformin on the adenosine A_1_ receptor signaling pathway in fat cells was explored. It was revealed that lipolysis triggered by DPCPX, an adenosine A_1_ receptor antagonist, was decreased in the presence of metformin. This proves that the anti-lipolytic activity of the tested drug is not solely restricted to adrenergic stimulation but also covers the adenosine A_1_ receptor signaling pathway. One of the key molecules common for both pathways and implicated in lipolysis regulation is intracellular cAMP. A rise in its concentrations is associated with increased TG hydrolysis, whereas a decrease evokes the opposite effect [6]. Previous studies have shown that metformin inhibits isoproterenol-induced lipolysis in rat adipocytes by diminishing the intracellular cAMP content and reducing PKA activity. These effects were found after 16-h exposure to the tested compound [33] and thus they may be related to changes in gene expression. Under physiological conditions, the intracellular cAMP level is subject to short-term regulation by alterations in the activities of adenylate cyclase (which catalyzes its synthesis) and phosphodiesterases (responsible for cAMP degradation) [6]. This short-term regulation is not associated with alterations in gene expression. To assess the relevance of cAMP in the mechanism of metformin action, lipolysis was induced by DB-cAMP, a cAMP analog that is not decomposed by intracellular phosphodiesterases. As mentioned above, the tested drug effectively restrained lipolysis stimulated by DB-cAMP. This indicates that a decrease in the intracellular cAMP pool [33] is not the main causative factor for the anti-lipolytic action of metformin.

Adipocyte lipolysis is influenced by numerous factors, including glucose [6,37]. It is known that the lipolytic process is diminished as a result of reduced intracellular glucose transport and/or metabolism [37,38]. Thus, the anti-lipolytic properties of metformin might be related to changes in the transport and/or metabolism of glucose. To verify this assumption, glucose was replaced by alanine. The intracellular transport of both compounds differs. Glucose enters adipocytes via GLUT1 (in the absence of insulin) or by GLUT4 (insulin-stimulated transport), whereas alanine reaches fat cells using the amino acid transport system A. Then, glucose and alanine, after glycolysis and deamination, respectively, are metabolized in mitochondria [39,40]. It was shown that metformin was capable of reducing epinephrine-stimulated lipolysis also under conditions of glucose deprivation, i.e., in the presence of alanine. This strongly suggests that its anti-lipolytic action is not due to inhibition of glucose transport but from alterations in mitochondrial metabolism. It is in line with data showing that in some kinds of cells, metformin reaches mitochondria and is an inhibitor of complex I in the electron transport chain. This effect is thought to be pivotal in the mechanism underlying metformin action at the cellular level. Braking of the electron transport chain in liver cells is associated with reduced hepatic glucose output. This effect is responsible for the blood-glucose-lowering action in metformin-treated patients [14,41,42]. The direct influence of metformin on the electron transport chain in freshly isolated adipocytes has not been elucidated. However, it is known that inhibition of this chain in fat cells by other agents (e.g., 2-bromopalmitate or rotenone) restrains the lipolytic process [37,43]. Hence, these results strongly suggest that metformin limits the lipolytic response of adipocytes via inhibition of the electron transport chain.

Adipocyte TG hydrolysis is associated with the formation of both glycerol and NEFA. The whole glycerol is released from fat cells, whereas a part of NEFA is released, a part is re-esterified, and the remaining amount undergoes oxidation. The proportion between these processes changes depending on various conditions [44,45]. Excessive lipolysis has pathological implications for the whole organism. The results of in vivo studies provided evidence that exaggerated glycerol release contributes to hyperglycemia and insulin resistance and plays a relevant role in the pathogenesis of type 2 diabetes. These pathological changes may, however, be prevented by inhibition of the lipolytic process [46,47]. Elevated blood levels of NEFA are also strongly associated with insulin resistance and type 2 diabetes, whereas lowering of NEFA exerts an opposite effect and improves tissue insulin sensitivity [48,49]. Therefore, we studied the effects of metformin on the release of both glycerol and NEFA. It was shown that epinephrine-stimulated release of glycerol and NEFA was substantially diminished in adipocytes exposed to the tested drug. The inhibitory effect of metformin was similar in the case of glycerol and NEFA. This indicates that metformin is an effective anti-lipolytic agent in rat adipocytes and is capable of decreasing the release of glycerol and NEFA.

Glycerol release from adipocytes is known to be elevated as a result of exposure to higher glucose levels [37,50]. To better elucidate metformin action, its influence on lipolysis was compared in the presence of low and high glucose levels. We showed that metformin markedly reduced epinephrine-stimulated glycerol release in the presence of 3 and 12 mM glucose. This is comparable to the inhibitory effect of insulin in adipocytes exposed to epinephrine and supraphysiological glucose levels [51]. Insulin is known to be the most effective physiological anti-lipolytic agent [6]. Although similar anti-lipolytic action was revealed for insulin and metformin, the mechanisms of their action are different. Insulin suppresses lipolysis predominantly by activating intracellular phosphodiesterases and the resulting cAMP decomposition [6], whereas metformin action covers inhibition of the electron transport chain. Metformin was also shown to be capable of reducing lipolysis triggered by DB-cAMP and DPCPX in the presence of 12 mM glucose. This indicates that the tested drug is a very effective anti-lipolytic agent in primary adipocytes and also exerts its action in the presence of a supraphysiological concentration of glucose.

Previous studies provided evidence that the anti-lipolytic effect of metformin in human adipocytes is dependent on AMP-activated protein kinase (AMPK) [24,31,32]. AMPK is a pivotal intracellular energy sensor implicated in regulating metabolic processes. Changes in its activity affect energy generation, depending on the current cellular demand. Apart from a relevant physiological role, AMPK is also a target for some drugs, including small molecule activators [52,53]. The involvement of AMPK in metformin action is in line with the results showing that induction of this enzyme by AICAR (a pharmacological activator) in rat adipocytes reduces the lipolytic response to epinephrine [37,45]. Moreover, it was revealed that both metformin and AICAR evoke similar effects on AMPK in human fat cells, i.e., phosphorylation at Thr172, and the resulting activation of the enzyme [31]. The relevance of AMPK in metformin action is additionally confirmed by the findings that its anti-lipolytic effect is suppressed in the presence of compound C (a pharmacological AMPK inhibitor) [31]. Moreover, AMPK1 α silencing in human adipocytes was shown to suppress metformin-induced phosphorylation of hormone-sensitive lipase (HSL) [24]. Thus, data on the effects of metformin on the mitochondrial electron transport chain and AMPK activity are fully coherent in the context of the anti-lipolytic properties. Inhibition of the chain results in intracellular AMP accumulation, which in turn activates AMPK and restrains the response of adipocytes to lipolytic stimulation [14,41].

Changes in glucose transport and metabolism strongly affect lactate release from fat cells. Lactate was recently recognized not only as a metabolic waste product but also as a relevant signaling molecule. This compound has been implicated in maintaining, among others, the whole-body energy balance, regulating redox homeostasis, and the metabolic activity of cells [27,54]. An excessive lactate output from fat cells has grave implications, such as impaired insulin sensitivity [30,55]. Given this data, the effects of metformin on lactate release from freshly isolated rat adipocytes were explored. Adipocyte exposure to a supraphysiological concentration of glucose was associated with an increased release of lactate compared with the effects elicited in the presence of low glucose. Metformin was, however, found to effectively decrease lactate release. A similar effect was observed in the presence of 3 and 12 mM glucose. Previous studies have shown that long-term (96-h) exposure of rat adipocytes to metformin augments lactate production. This stimulatory action was, however, revealed in the presence of insulin [56]. The ability of the tested drug to reduce lactate output shown in the present research may be attributed to changes in AMPK activity. Metformin was demonstrated to activate AMPK in adipocytes [31], and activation of this enzyme by AICAR decreases lactate release from fat cells [37]. AMPK induction is also associated with reduced glucose uptake and oxidation in isolated adipocytes [37,57,58]. Thus, the mechanism whereby the tested compound limits lactate production is similar to the suppression of lipolysis, i.e., inhibition of the electron transport chain, rise in AMP content, activation of AMPK, and the resulting diminished glucose conversion to lactate.

**Figure 9:**
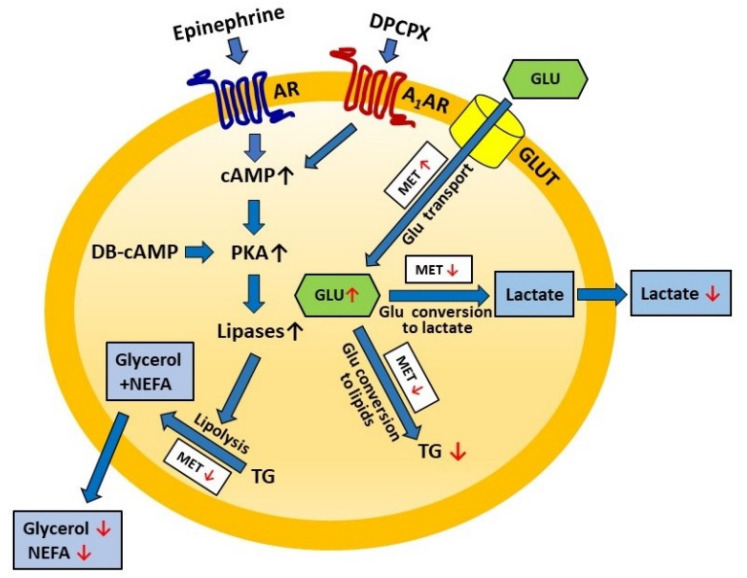
Schematic representation of metformin action on adipocyte lipolysis and lactate release. DPCPX—adenosine A_1_ receptor antagonist; AR—adrenergic receptor; A_1_AR—adenosine A_1_ receptor; DB-cAMP—dibytyryl-cAMP (cAMP analog); PKA—protein kinase A; TG—triglycerides; NEFA—non-esterified fatty acid; GLU—glucose; GLUT—glucose transporter; MET—metformin; red arrow—metformin effect.

Figure 9 presents the effects of metformin on lipolysis and lactate release shown in the present study. The concentrations of metformin and time of exposure in various in vitro studies are very differentiated [13]. In our study, adipocytes were exposed for 4 h to 1 mM metformin. Such a term of treatment is short, and the concentration is relatively low compared with research on preadipocyte maturation or gene expression [16,17,18,24,32]. However, the effects elicited by the tested drug were quite large. In in vitro study, the concentrations of the tested compounds are usually much higher compared to their blood levels. The concentration of metformin used in the present research was also higher compared to the blood levels of the drug after intragastric treatment. However, prolonged metformin administration is associated with its increased tissue accumulation. Due to a cationic nature, metformin accumulates especially in the mitochondria, and the mitochondrial metformin content may even be many times higher than in blood. Moreover, mice studies have shown that per oral metformin administration resulted in its liver peak concentration being higher than in our study, reaching 1500 µM (1.5 mM), while blood levels were about 50 µM [59]. This justifies the use of the 1 mM metformin concentration in the present study. Moreover, the preliminary experiments showed that a 1 mM concentration of metformin induces (in our experimental conditions, i.e., freshly isolated cells and 4-h exposure) a clear-cut and repetitive adipocyte response to metformin. The tested drug used at a lower concentration (0.5 mM) was less effective. Moreover, in some previous studies, 0.5 [32] or 2 mM [31] metformin was used. However, in our preliminary experiments, metformin at the 1 mM concentration evoked a marked inhibitory effect on lipolysis and lactate release. Thus, higher concentrations were not taken into account. Additionally, we showed that formazan formation from MTT, reflecting cell viability, was not significantly affected in the presence of metformin. Thus, it can be supposed that the effects of metformin revealed in this study are not related to reduced adipocyte viability.

## 4. Materials and Methods

### 4.1. Reagents

Metformin, D-glucose, L-alanine, bovine serum albumin (BSA, fraction V), collagenase (from *Clostridium histolyticum*), epinephrine, insulin, dibutyryl-cAMP (DB-cAMP), 8-Cyclopentyl-1,3-dipropylxanthine (DPCPX), 3-(4,5-dimethylthiazol-2-yl)-2,5-diphenyltetrazolium bromide (MTT), dimethyl sulfoxide (DMSO), heptane, silicone oil, phloretin, and all reagents used to prepare Krebs-Ringer buffer (118 mM NaCl, 4.8 mM KCl, 1.3 mM, CaCl_2_, 1.2 mM KH_2_PO_4_, 1.2 mM MgSO_4_, 24.8 mM, NaHCO_3_) were obtained from Sigma-Aldrich (St. Louis, MO, USA).

Reagents for glycerol assay: isopropanol, anhydrous ammonium acetate, sodium metaperiodate, and 2,4-pentadione were purchased from Sigma-Aldrich (St. Louis, MO, USA), and glacial acetic acid was obtained from POCH (Gliwice, Poland).

Reagents used for the determination of non-esterified fatty acids (NEFAs): triethanolamine, acetic acid, and diethyldithiocarbamate were also purchased from Sigma-Aldrich (St. Louis, MO, USA), whereas butanol-1 and chloroform were obtained from POCH (Gliwice, Poland).

Lactate was measured using lactate dehydrogenase, NAD^+^, and glycine buffer (Sigma-Aldrich, St. Louis, MO, USA). Glucose D-[^14^C(U)], 2-deoxy-D-glucose-[1-^14^C], and scintillation cocktail-OptiPhase HiSafe were purchased from Perkin Elmer (Boston, MA, USA).

### 4.2. Animals

In the experiment, fat tissue was taken from male Wistar rats weighing 280–300 g. Rats were purchased from Mossakowski Medical Research Centre Polish Academy of Sciences in Warsaw (Poland). After the supply, animals were subjected to at least a two-week adaptation period. Rats were maintained in cages in an air-conditioned animal room with a constant temperature (21 °C) and dark/light cycle (12/12 h). Animals were fed ad libitum a standard laboratory diet (Labofeed B, “Morawski”, Kcynia, Poland), and had free access to the drinking water. Rats were used only for tissue sampling (any experiments on live animals were not performed); therefore, the agreement of the Local Ethical Commission for Investigations on Animals was not required.

### 4.3. Adipocyte Isolation

Adipocytes were isolated according to the method described by Rodbell [59] with some modifications [60]. After decapitation of rats, epididymal fat tissue was taken, rinsed with 0.9% NaCl, placed in a plastic flask, and cut down with scissors. Then, the tissue was incubated in Krebs-Ringer buffer (KRB) containing 3 mM glucose with collagenase (1 mg/mL). Before use, the buffer was gassed with a mixture of O_2_ and CO_2_ (95% and 5%, respectively), and its pH was adjusted to 7.4 with 0.1 M NaOH. The tissue was incubated in a water bath at 37 °C with gentle shaking for about 60 min. After this time, cells were filtered through a nylon mesh and were washed a few times with the buffer containing no collagenase. Then, adipocytes were placed in polystyrene tubes and left for flotation. Afterward, cells were mixed with the appropriate volume of KRB, and the aliquots of adipocyte suspensions were used in the experiments with metformin.

### 4.4. Effects of Metformin on Lipogenesis

To determine the effects of metformin on glucose conversion to lipids (lipogenesis), adipocytes were incubated in the KRB (pH = 7.4) containing 3 mM glucose, 0.5 μCi of glucose D-[^14^C(U)], 3% BSA, and 10 mM HEPES. Basal lipogenesis was studied without any hormone, whereas stimulated lipogenesis was determined in the presence of 10 nM insulin. To explore the influence of metformin on insulin-stimulated lipogenesis, adipocytes were exposed to insulin and 1 mM metformin. In each case, 10^6^ adipocytes were maintained for 4 h in 1 mL of KRB at 37 °C with gentle shaking. After the end of the incubations, the reaction was stopped by the addition of Dole’s extraction mixture (isopropanol: heptane: 0.5 M H_2_SO_4_; 40:10:1). After shaking, 2 mL of H_2_O and 3 mL of heptane were added. Then, the tubes were shaken once again, samples of the upper phase were transferred into counting vials containing the scintillation cocktail (OptiPhase HiSafe 3, PerkinElmer), and total lipid radioactivity was measured using a β-counter.

### 4.5. Effects of Metformin on Glucose Transport

The glucose transport into adipocytes was measured using the non-metabolizable glucose analog (2-deoxy-D-glucose). Suspensions containing 10^6^ cells/mL/tube were preincubated with 0.5 mM glucose without metformin or with 1 mM metformin for 10 min at 37 °C with gentle shaking. Then, 10 nM insulin was added to each tube. The additional group of adipocytes was preincubated with 0.5 mM glucose without insulin as a basal one. After the next 20 min, 1 μCi 2-deoxy-D-glucose-[1-^14^C] was added and the tubes were incubated once again for 3 min. To stop the reaction, 400 μL of the ice-cold KRB with 3 mM phloretin was used. Then, 400 μL of silicone oil was added to separate the cell suspensions from the buffer, and tubes were centrifuged. The upper phase with cell suspensions was transferred into the vials with the scintillation cocktail, and the radioactivity was measured using a β-counter.

### 4.6. Effects of Metformin on Lipolysis

To study basal lipolysis, isolated adipocytes were maintained in KRB without lipolytic stimuli. In the present study, the effects of metformin on lipolysis were compared in the presence of low and high glucose concentrations. Apart from various glucose levels, lipolytic agents that differed in their mechanism of action were used.

In the first part of the experiment, epinephrine (physiological β-adrenergic agonist) was added to stimulate lipolysis. To study the effects of metformin on lipolysis under conditions of a low concentration of glucose, adipocytes were incubated in KRB containing 3 mM glucose; 3 mM glucose and epinephrine; or 3 mM glucose, epinephrine, and metformin. Isolated cells were also exposed to 12 mM glucose; 12 mM glucose and epinephrine; or 12 mM glucose, epinephrine, and metformin. In each case, fat cells were maintained for 3 h without epinephrine. Then, 0.5 µM epinephrine was added and cells were incubated for an additional 1 h.

In the next set of our research, epinephrine was replaced by DB-cAMP (a direct activator of protein kinase A). In the experiments with the low glucose concentration, isolated adipocytes were exposed to KRB containing 3 mM glucose; 3 mM glucose and DB-cAMP; or 3 mM glucose, DB-cAMP, and metformin. To study the effects of metformin on DB-cAMP-induced lipolysis in the presence of high glucose amounts, adipocytes were exposed to 12 mM glucose; 12 mM glucose and DB-cAMP; or 12 mM glucose, DB-cAMP, and metformin. Adipocytes were preincubated for 3 h without DB-cAMP. After this time, 0.5 mM DB-cAMP was added, and incubations were performed for the next 1 h.

In our study, DPCPX (an adenosine A_1_ receptor antagonist) was also used as a lipolytic agent. In the experiments with DPCPX, the effects of metformin on lipolysis were also compared in the presence of low and high glucose levels. For this purpose, isolated cells were incubated in KRB with 3 mM glucose; 3 mM glucose and DPCPX; or 3 mM glucose, DPCPX, and metformin. Moreover, fat cells were exposed to 12 mM glucose; 12 mM glucose and DPCPX; or 12 mM glucose, DPCPX, and metformin. In each case, adipocytes were preincubated for 3 h without DPCPX. Following preincubation, 0.5 µM DPCPX was added, and fat cells were subjected to the next 1-h incubation.

Apart from the experiments with glucose, the effects of metformin on epinephrine-stimulated lipolysis were also studied under conditions of glucose deprivation. For this purpose, isolated adipocytes were incubated in KRB containing 12 mM alanine; alanine with epinephrine; or alanine, epinephrine, and metformin. Similar to the studies with glucose, adipocytes were preincubated for 3 h without epinephrine. After the pre-exposure, 0.5 µM epinephrine was added followed by 1-h cell incubation.

In each study addressing lipolysis, 10^6^ adipocytes were maintained in 1 mL of KRB at 37 °C with gentle shaking. After the end of the incubations, fat cells were removed by aspiration, and aliquots of KRB were taken to determine glycerol and, in some cases, also non-esterified fatty acids (NEFAs).

### 4.7. Glycerol Determination

The concentration of glycerol in the incubation medium was measured according to the method described by Foster and Dunn [61] with some modifications [60]. First, the samples were deproteinized in Eppendorf tubes using 10% trichloroacetic acid (TCA). For this purpose, tubes were vortexed and centrifuged, and the supernatant was taken for further analysis. In our study, the colorimetric Hantzsch condensation method was used. In this method, glycerol, in the presence of sodium meta-periodate, is oxidized to formaldehyde. Then, formaldehyde reacts with ammonia and acetylacetone to give a yellow product (3,5-diacetyl-1,4-dihydrolutidine). The color intensity reflects the concentration of glycerol. Finally, the absorbance of each sample was read at 410 nm.

### 4.8. Non-Esterified Fatty Acid Determination

The content of NEFAs in the incubation medium was detected according to the method described by Duncombe [62] with some modifications [63]. First, aliquots of the buffer were mixed with chloroform and with copper reagent (containing 1 M triethanolamine: 1 M acetic acid: 6.5% Cu(NO_3_)_2_; 9:1:10). After vortexing, samples were centrifuged. Then, the upper phase was removed, and aliquots of the lower phase were transferred to new tubes. Finally, diethyldithiocarbamate reagent (0.1% butanol solution) was added to each tube and after shaking, the absorbance was measured at 440 nm.

### 4.9. Effects of Metformin on Lactate Release

To study lactate release, freshly isolated adipocytes (10^6^ cells per mL) were maintained in buffer containing 3 or 12 mM glucose without metformin or in the presence of 1 mM metformin. Fat cells were incubated for 4 h at 37 °C with gentle shaking. After the end of the incubations, the lactate concentrations in the buffer were measured.

### 4.10. Lactate Determination

To determine the lactate concentrations in the incubation medium, adipocytes were removed and aliquots of KRB were mixed with 10% trichloroacetic acid to remove proteins. Then, tubes were centrifuged, and the supernatant was taken for analysis. The enzymatic method with lactate dehydrogenase was used [64]. According to this method, lactate in the presence of NAD is converted into pyruvate and NADH. The reaction is catalyzed by lactate dehydrogenase. The samples were mixed in tubes containing glycine buffer, lactate dehydrogenase, and NAD. Then, the tubes were incubated at 37 °C for 15 min. After the incubations, samples were cooled, and the absorbance of NADH generated from NAD^+^ was read at 340 nm. The intensity of absorbance reflects the concentration of lactate in each sample. The method originally described for lactate determination [64] was modified; NAD was used instead of 3-acetylpyridine diphosphopyridine nucleotide, 0.6 M glycine buffer was used instead of borate, samples were deproteinized, and the absorbance was read at 340 nm instead of 363 nm.

### 4.11. Effects of Metformin on Adipocyte Viability

Fat cell viability was determined using the MTT test. In this method, MTT in living cells is converted to formazan. The MTT test is commonly used for the determination of cell viability and is suitable for this kind of research [65]. Isolated adipocytes (10^6^ per mL) were first incubated for 4 h in KRB containing 3 mM glucose alone or glucose in the presence of 1 mM metformin. Then, all cells were washed with the buffer without metformin. This was followed by adipocyte incubation in buffer containing 3 mM glucose and 0.5 mg/mL MTT. After 1-h incubation, 1 mL of isopropanol was added to each sample, and tubes were shaken and centrifuged. All incubations were performed at 37 °C with gentle shaking. Finally, the absorbance of formazan was measured at 560 nm.

### 4.12. Statistical Analysis

The obtained results represent the mean ± standard error of the mean (SEM) from 3 independent experiments in 5 repetitions (*n* = 15). The results were statistically evaluated using analysis of variance and Tukey’s multiple range test. Data analysis was performed using the GraphPad Prism for Windows software (license no. GRA/3802/2015, La Jolla, CA, USA). The differences were considered statistically significant at *p* < 0.05.

## 5. Conclusions

In conclusion, metformin was shown to decrease lipogenesis and increase glucose transport in isolated rat adipocytes. The tested compound was also shown to reduce the lipolytic response of adipocytes to physiological and pharmacological stimuli. The drug is effective in the presence of physiological and supraphysiological glucose levels. Metformin was also demonstrated to decrease lactate release from adipocytes. It can be supposed that the influence on lipolysis and lactate output is due to the known inhibitory effects of metformin on the mitochondrial electron transport chain. Our results show that short-term exposure to metformin affects the metabolism of primary rat adipocytes.

## Data Availability

All raw data are available to the corresponding author upon reasonable request.

## Sample Availability

Samples of the compound are not available from the authors.
